# 0093. Mitochondrial function of immune cells in severe sepsis and septic shock - a prospective observational cohort study

**DOI:** 10.1186/2197-425X-2-S1-P5

**Published:** 2014-09-26

**Authors:** TM Merz, AJ Pereira, V Jeger, JM Stephan, T Jukka, S Djafarzadeh

**Affiliations:** University Hospital Bern, Department of Intensive Care Medicine, Bern, Switzerland

## Introduction

Circulating immune cells contribute to sepsis pathophysiology. Immune system activation increases cell energy requirements [1]; impaired mitochondrial function and ATP production may modulate the immune response in sepsis.

## Objectives

To investigate mitochondrial enzyme activities and ATP content of monocytes, B cells and CD4^+^ T cells in patients with severe sepsis and septic shock.

## Methods

30 patients with severe sepsis or septic shock were studied at ICU admission and after 24 and 48 hours. Immune cells were identified and isolated using an immunomagnetic positive cell isolation procedure (Dynabeads® for Human Monocytes, Human B cells, Human CD4 cells, Invitrogen Dynal AS, Oslo, Norway). Enzymatic activity of mitochondrial complexes I, IV, and ATP synthase and ATP content were measured spectrophotometrically and expressed as ratio to citrate synthase for enzymatic activities to account for mitochondrial mass and as mass per µg of cellular protein for ATP content. Maximal mitochondrial enzymatic activities and ATP in the first 48h of sepsis were compared with samples from 20 healthy volunteers (Mann Whitney test).

## Results

**Figure 1:**
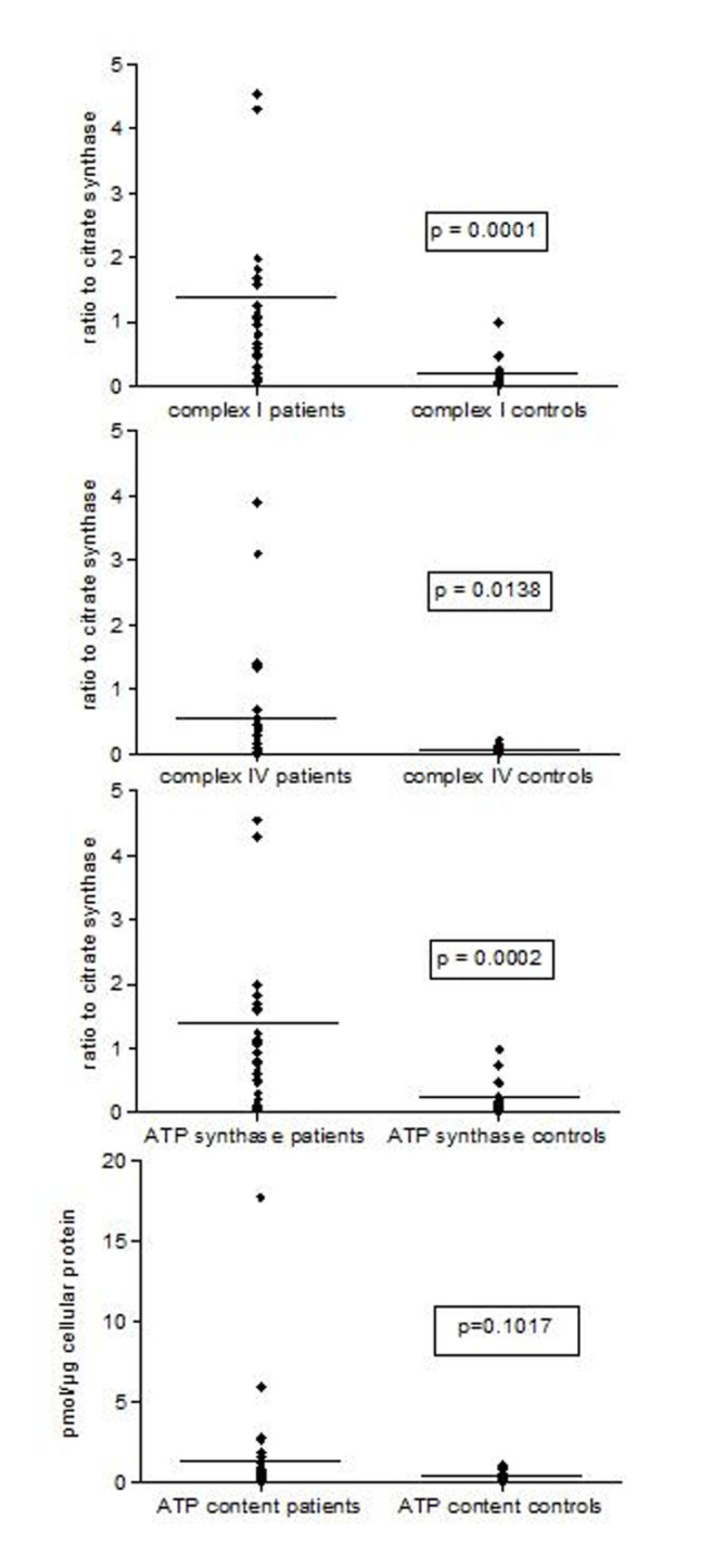
Maximal mitochondrial enzymatic activities and ATP content of human immune cells in the first 48h of sepsis : Monocytes

**Figure 2:**
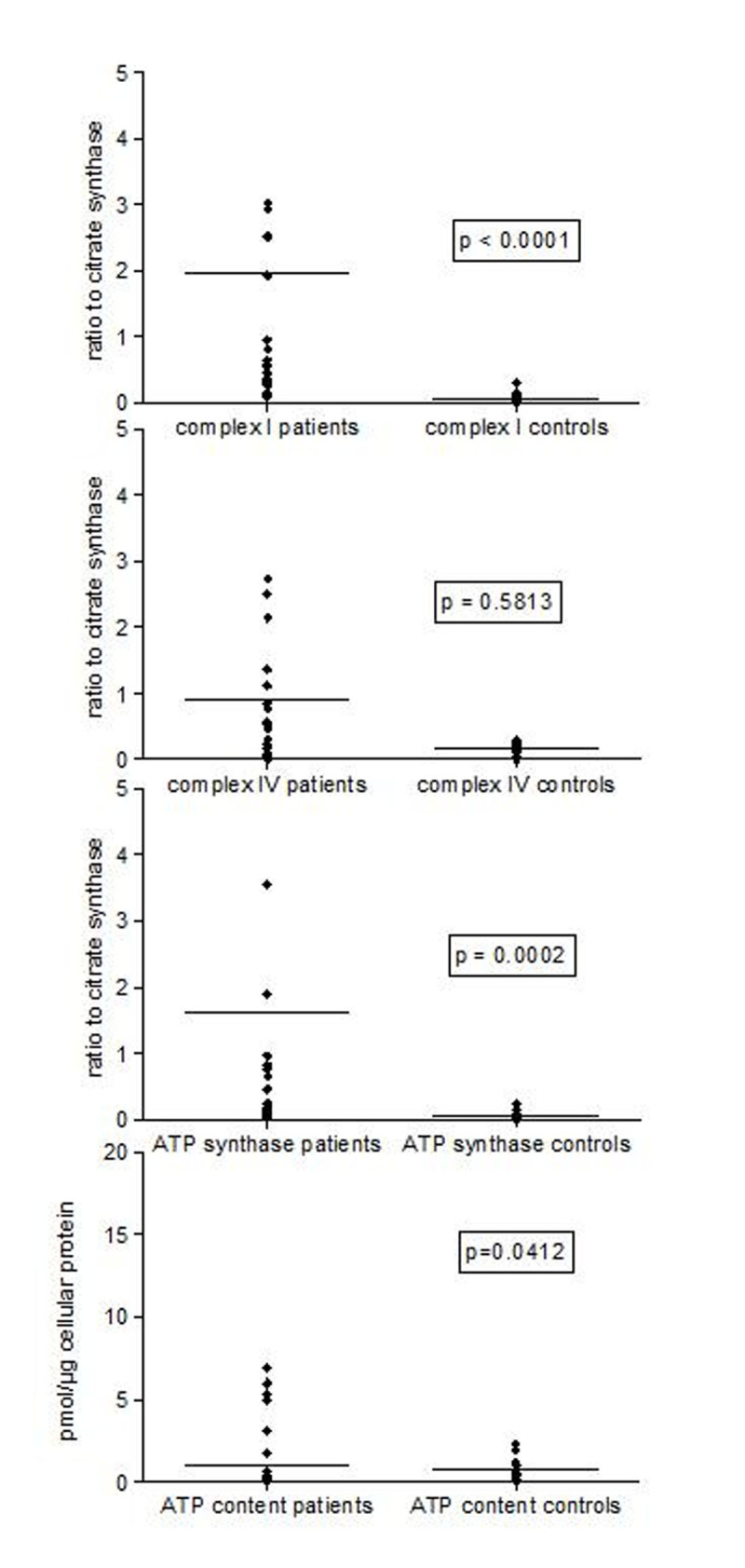
Maximal mitochondrial enzymatic activities and ATP content of human immune cells in the first 48h of sepsis: B cells

**Figure 3:**
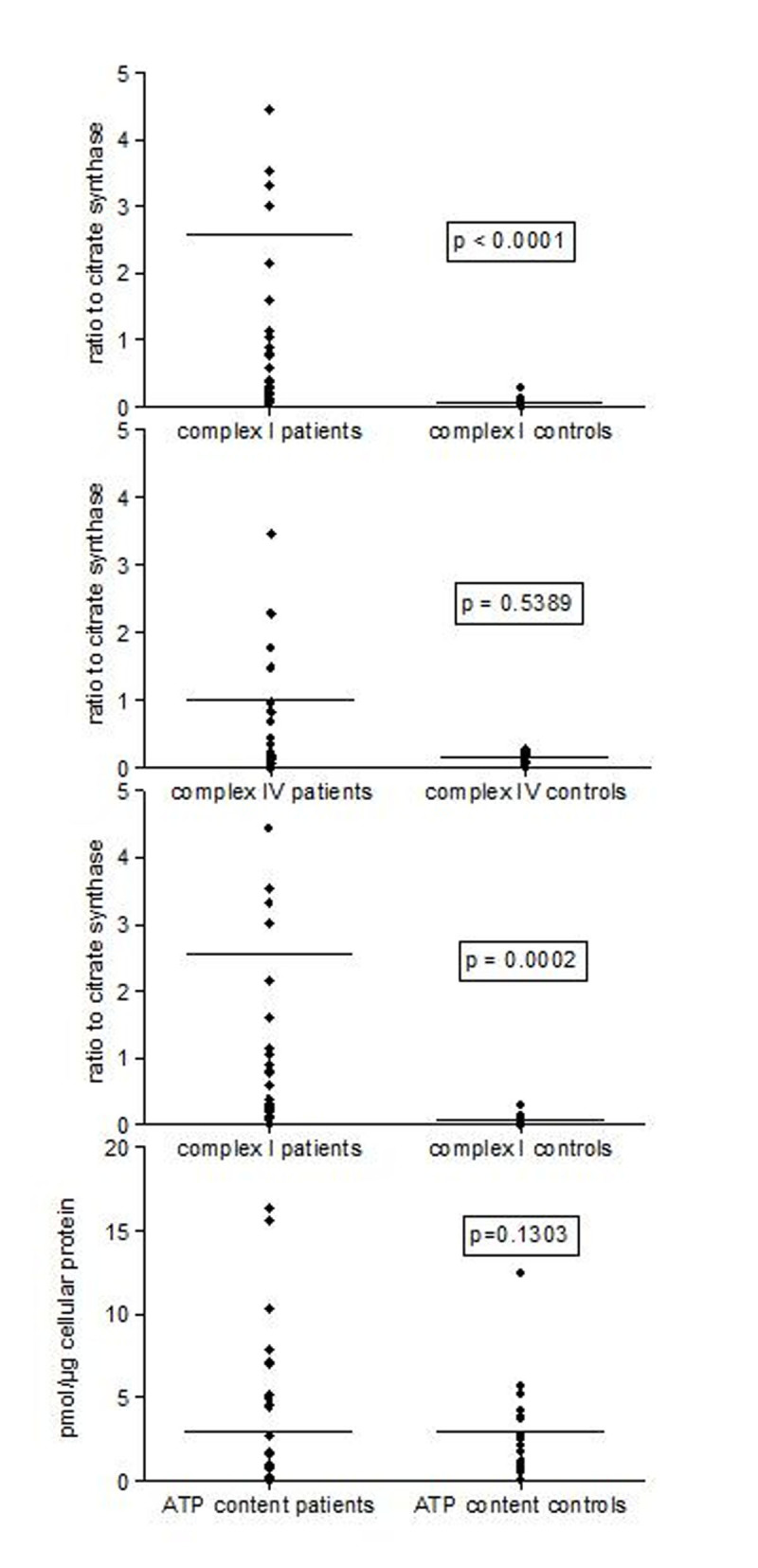
Maximal mitochondrial enzymatic activities and ATP content of human immune cells in the first 48h of sepsis: T cells

Complex I and ATP synthase activities in sepsis were increased (p< 0.0001 to 0.0002; Figure 1, 2, 3). Complex IV activity was increased in monocytes (p=.0138). Complex IV activity in B and T cells was highly variable (Figures 1a-c). ATP content was increased in B cells but not in monocytes or T cell in sepsis compared to control. There were no differences between survivors and non-survivors in any of the enzymatic activities or in ATP content.

## Conclusion

Mitochondrial enzyme activities of human immune cells are increased in severe sepsis and septic shock. We suggest that this helps to preserve normal or increased cell ATP content in acute inflammation.
